# Hexagonal Bismuthene and Bismuth Nanoparticles for Light-To-Heat Conversion

**DOI:** 10.1021/acsami.5c11661

**Published:** 2025-08-28

**Authors:** Marta Alcaraz, Pau Congost-Escoin, Christian Dolle, Josep Canet-Ferrer, Gonzalo Abellán

**Affiliations:** Instituto de Ciencia Molecular (ICMol), 16781 Universidad de Valencia, 46980 Paterna, Spain

**Keywords:** bismuthene, bismuth nanoparticles, light-to-heat conversion, Raman thermometry, surface plasmon resonance calculations, gold nanoparticles

## Abstract

Bismuth is an inexpensive, biocompatible, and semimetallic material. Light absorption in bismuth occurs by means of intraband transitions from mid-IR to UV due to its exotic electronic structure. Thus, bismuth has been pointed out as an excellent material in applications relying on optical absorption, such as computed tomography, photocatalysis, and photothermal therapy. Herein, we present an extensive study of the unexplored thermo-optical response of high-quality bismuth nanomaterials: hexagonal bismuthene (hBi) and spherical bismuth nanoparticles (sBiNPs). We observed the light-to-heat conversion (LHC) performance of both compared to that of functionalized Au NPs (NPs) as an alternative to plasmonic NPs for LHC applications. As a result, we have illustrated the potential of bismuth NPs and bismuthene for thermo-optical applications, demonstrating the potential of bismuth NPs and bismuthene for thermo-optical applications, with precise local heating quantified via Raman thermometry. Under 2.1 mW excitation at 633 nm, maximum temperatures of 425 K for AuPMBT, 400 K for sBiNPs, and 325 K for hBi were reached. These values highlight the competitive LHC efficiency of bismuth-based nanostructures, especially sBiNPs, and reveal the influence of thermal diffusion in 2D systems like hBi.

## Graphical abstract

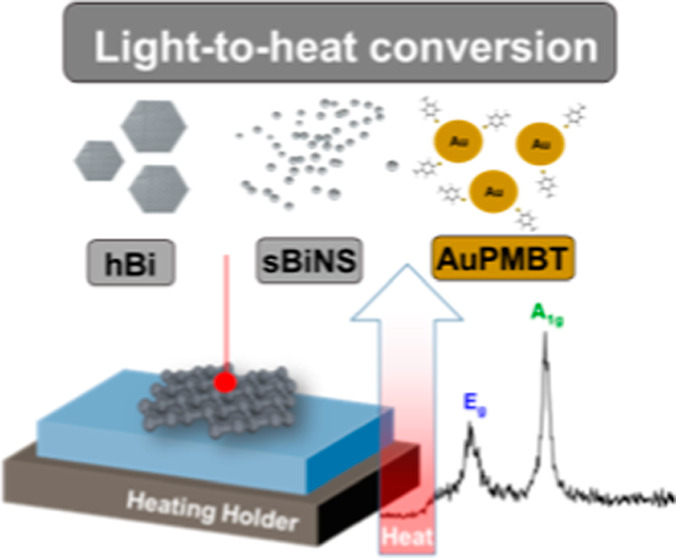

## 1 Introduction

A few decades ago, the interest in plasmonic nanoparticles (NPs) as light-driven heat sources has been growing slowly but surely.^1–4^ The optical absorption is enhanced in the presence of localized surface plasmon resonances (LSPRs) with the corresponding photoinduced temperature variations.^5–7^ This ability to generate heat at the nanoscale has been exploited in various applications, including biosensing, photothermal therapy, and photocatalysis, among others.^4,5,8–12^ In particular, applications necessitating resistive loss, such as photothermal therapy or photocatalysis, are mainly satisfied by Au and Ag NPs.^13–16^ Nevertheless, the light-to-heat conversion (LHC) in LSPR is bound to the nonradiative decay of photogenerated carriers at the conduction band and in the case of the extensively used Au and Ag NPs, this could be a drawback due to their electronic structure which favors the interband absorption.^17^ To overcome this drawback, many other nanomaterials beyond noble metals have been explored. For instance, compounds consisting of III–V semiconductors (GaAs or InP), nitrides (GaN, TiN, ZrN), transparent conductive oxides (ZnO and ITO), and 2D materials (graphene and MoS_2_). The optimization of these unconventional materials could enhance the performance of the conventional ones, overcoming their limitations^17–19^ .

**Scheme 1:**
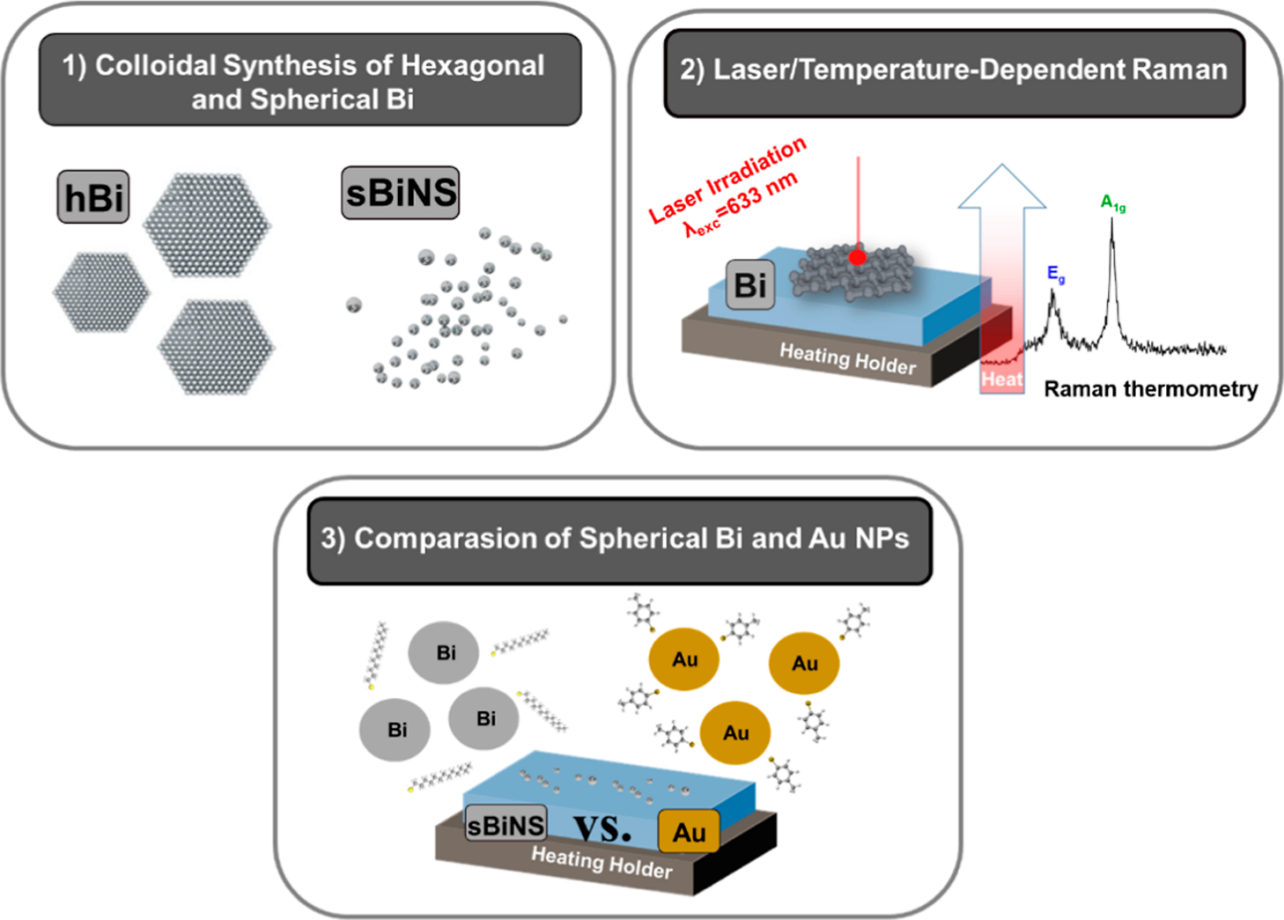
Scheme Summarizing the (1) Synthesis of Hexagonal Bismuthene and Spherical Bi NPs through a Colloidal Approach; (2) In Addition, Raman Thermometry Experiments of Hexagonal Bismuthene and Spherical Bi Were Performed; and (3) Finally, a Plausible Comparison Was Performed Using Functionalized Au NPs with 4-Methylbenzenethiol

Since the observation of plasmons in graphene, atomically thin 2D materials have been explored for plasmonic applications. Two-dimensional plasmonic materials have gained great interest due to their high tunability, broad doping range, and favorable depolarization factors, allowing better control over their surface plasmon responses.^17,20–22^ Among these 2D materials, a new family known as 2D-Pnictogens (P, As, Sb, Bi) has emerged, showcasing exceptional and customizable physicochemical properties for their application in optoelectronics, electromagnetic systems, energy storage, catalysis, sensing, and biomedicine.^23–26^ In particular, Bi presents itself as a biocompatible and inexpensive metal with an exotic electronic structure that enables the possibility of confined light at its localized surface plasmon resonances in a broad range of the spectrum. Furthermore, Bi is an alternative to Au as a contrast agent for imaging in X-ray computed tomography, with demonstrated capabilities for photothermal therapy.^27–30^ Moreover, Au NPs have precisely a larger mass concentration for operation resulting in toxicity, above all reckoning with the long retention time of Au NPs.^31,32^ Furthermore, Bi has a semimetallic character capable of supporting plasmonic effects from the mid-IR to the UV region, mainly induced by intraband transitions, which is of particular interest in photocatalysis.^27,33,34^ As an example, several Bi based photocatalytic systems have been reported for oxidation of NO without any toxic intermediates.^35^

Furthermore, the surface-to-volume ratio of a material plays a critical role in determining its photocatalytic efficiency, as the higher ratio of 2D materials allows for more active sites where photocatalytic reactions can occur, enhancing their ability to use light energy to drive chemical reactions. Consequently, materials designed with higher surface-to-volume ratios exhibit superior photocatalytic performance and more effective degradation of pollutants and conversion of solar energy into chemical energy.^36,37^

In this work, we demonstrate the potential of hexagonal bismuthene (hBi) as an alternative for application in LHC. Their thermo-optical properties have been studied by means of Raman thermometry (Scheme 1). Continuous laser exposure of hBi at progressively higher power levels induces a clear linear dependence of the temperature increase. However, the hexagonal flat morphology of hBi prevents proper benchmarking of the resistive loss in Bi. For this reason, the temperature increase around hBi has been compared with that in spherical Bi (sBiNPs) and functionalized Au with 4-methylbenzenethiol (AuPMBT) NPs with diameters of 50 and 20 nm, respectively. From this comparison, we can conclude that the light absorption and LHC efficiency are comparable in the case of Au and sBiNPs. This study proves the potential of Bi as a viable alternative for local heating applications, particularly in scenarios where the chemical properties of Au may pose a technical challenge. In this situation, the moderate response of hBi is attributed to a better thermal contact of the hexagonal geometry (with respect to the spherical one) and the corresponding higher heat diffusion. Our findings highlight the potential of Bi for photothermal applications in terms of light absorption in addition to its advantages in terms of radiosensitivity and toxicity. This opens new avenues for the utilization of Bi nanomaterials in medical and technological applications where controlled heat generation is crucial.^38–40^

## 2 Synthetic Methods

### 2.1 Colloidal Synthesis of Spherical Bi NPs (sBiNPs) with Diameters of 55 ± 10 nm

Spherical Bi NPs (sBiNPs) were synthesized from a 100 mM stock solution of bismuth neodecanoate in 1-octadecene, using similar conditions reported previously.^41^ For the colloidal synthesis, 1.25 mL (1 equiv) of the stock solution was mixed with 22.22 mL of 1-octadecene and 1.23 mL (30 equiv) of oleylamine (>98%) in a 100 mL two-neck flask. The flask was evacuated and heated in an oil bath at 100 °C under stirring with continuous illumination by commercial white light LED (Philips), after which the atmosphere was changed to Ar and the mixture was stirred for another 2 min. The volume of 0.3 mL (10 equiv) of 1-dodecanethiol (>98%) was injected through a septum and the mixture was stirred until a color change from yellow to black was observed. The reaction time varied from 1 to 2 min. Afterward, the reaction was quenched in an ice bath, then subjected to centrifugation (10 min, 10,000 rpm) and redispersion/washing cycles (3 times) in chloroform inside an Ar-filled inert gas glovebox. All the chemicals were purchased from Sigma-Aldrich.

### 2.2 Colloidal Synthesis of Spherical Bi NPs (sBiNPs) with Diameters of 140 ± 40 nm

The sBiNPs with diameters of 140 ± 40 nm were synthesized following the procedure already explained for the sBiNP of 55 ± 10 nm, varying only the reaction time from 3 to 4 min.

### 2.3 Colloidal Synthesis of Hexagonal Bismuthene (hBi)

The hexagonal bismuthene (hBi) was synthesized following the procedure already reported in our group,^41^ setting a temperature of 200 °C for a duration of 20 s.

### 2.4 Au NP Functionalization

Au NPs of 20 nm were modified using a method already described with 4-methylbenzenethiol (PMBT) from Sigma-Aldrich (98%).^42^ Au NPs (0.025 mmol, 1 equiv) were redispersed in 4 mL of a mixture of 1:1 H_2_O/EtOH with PMBT (0.2 mmol, 8 equiv). The solution was stirred overnight. The AuPMBT NPs were cleaned and centrifuged with ethanol (2 mL twice). Finally, the Au NPs were redispersed in fresh ethanol for storage. Samples for further characterization were prepared from the suspension in ethanol and chloroform (TEM, Raman, DLS).

## 3 Experimental Section

### 3.1 Morphological and Spectroscopy Characterization

The morphologies of both hBi and sBiNPs were determined by TEM and SEM images. To better establish the nanoparticle size, counting statistics from TEM images were carried out (Figures 1, S5–S6). Figure 1a,b shows hBi, which exhibits a perfect hexagonal shape with a lateral size close to a micrometer. The hexagonal NPs are extremely flat with lateral dimensions of a few hundred of nanometers, (ca. 420 nm)^41^ with an average lateral size of 760 ± 140 nm, as the statistical analysis of more than 50 nanosheets reveals (Figure S5).

**Figure 1:**
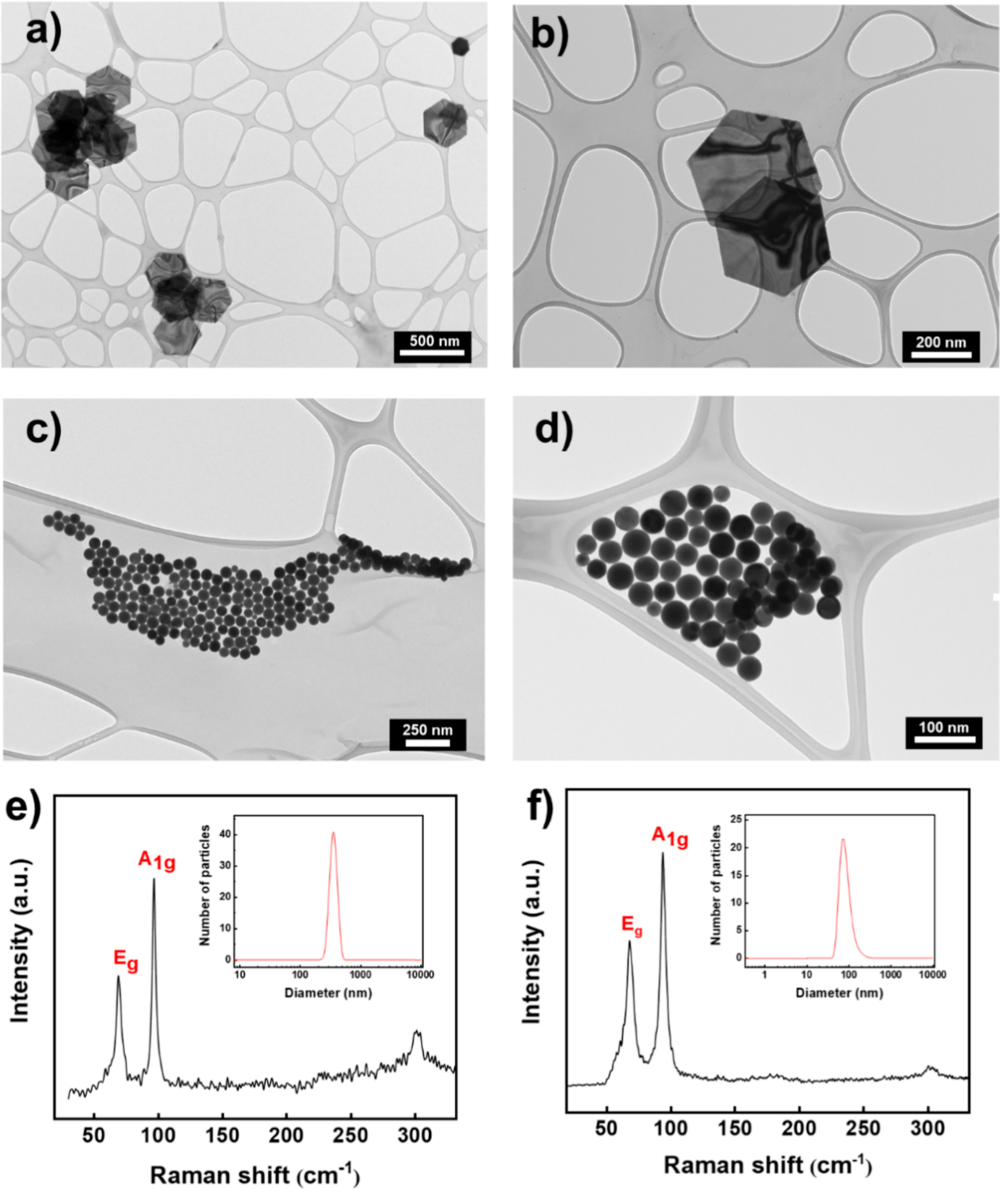
Morphological and spectroscopy characterization of hexagonal and spherical Bi NPs. (a) Overview TEM image of hBi. (b) Enlarged TEM image of hBi. (c) Overview TEM image of sBiNPs. (d) Enlarged TEM image of sBiNPs. (e) Raman spectra of hBi at room temperature, with DLS measurement shown in the inset. (f) Raman spectra of sBiNPs at room temperature with DLS measurement shown in the inset.

On the other hand, sBiNPs exhibit a spherical shape with diameters of 55 ± 10 nm (Figure 1c,d and S6a,b). The morphological characterization is completed with Dynamic Light Scattering (DLS) measurements; see the results in the Supporting Information (Figure S1). Figure S1 estimated average hydrodynamic radius of sBiNPs (Figure S1b) is within the range of 42–200 nm (maximum 80 nm), whereas hBi (Figure S1a) exhibited an estimated hydrodynamic radius from 310–440 nm (maximum 369 nm). As the changes in morphology, size, and aggregation could affect the thermo-optical properties of the nanomaterial,^43,44^ we optimized the procedure to transfer our NPs on the silicon surface, to reduce the size and number of aggregates. Furthermore, we performed XRD of the synthesized Bi NPs (Figure S2), revealing that despite their differences in shape and size, both NPs have the same crystalline structure, showing the metallic bismuth hexagonal structure with high degree of crystallinity.^41^ Interestingly, it reveals the preferential orientation of the deposited 2D hBi, as planes (003) at 22.47° and (006) at 45.86°, related to the interlayer distances, are more intense than for the sBiNPs, which shows no preferential orientation when deposited due to their round shape.

A commercial Raman setup operating at a 633 nm wavelength was used to study the vibrational spectra of hBi and sBiNPs at room temperature. The Raman spectra of sBiNPs in Figure 1f reveal the presence of the fundamental vibrations, in particular the modes E_g_ and A_1g_ at 69 and 93 cm^–1^, respectively. These Raman shifts are similar to the ones observed for the Bi powder, which are 69 cm^–1^ for E_g_ and 94.5 cm^–1^ for A_1g._^45^ Nevertheless, the Raman modes for the hBi in Figure 1e present a blue shift in both vibrational modes E_g_ and A_1g_, as compared with the sBiNPs (Figure 1f). The A_1g_ peak shifted a few units: from 93 cm^–1^ observed in sBiNPs to 97 cm^–1^ in Raman spectra of hBi, and the E_g_ peak shifted slightly, from 68 to 69 cm^–1^. The noticeable blue shift can be attributed to the layer-dependence of the vibrational modes, as occurred to graphene and other multilayer 2D materials stacked by van der Waals interactions.^46,47^

### 3.2 Raman Thermometry

Under laser irradiation, the temperature of the NPs could be considerably increased, resulting in a change in the Raman spectra. Hence, Raman thermometry is used to monitor the NP temperature as an estimation of the local temperature in the NP surroundings.^48–51^ For the purpose of studying the LHC of Bi based nanomaterials first, we conducted temperature-dependent Raman spectroscopy of hBi (Figure 2a). The experiment was carried out in an inert atmosphere heating holder, allowing accurate temperature control over the samples under study between 293 and 503 K (further details in the Supporting Information). For this purpose, the sample was excited with 0.293 mW at λ_exc_ = 633 nm. In these conditions, the heat generated by the excitation laser is negligible, allowing a reversible and reproducible temperature dependence. This is important to avoid undesired artifacts due to oxidation or overheating, as observed when exciting with green lasers or red lasers at higher powers (Figures S3 and S4). In Figure 2b, a clear decrease in the intensity and broadening as the temperature is increased. These effects are accompanied by an almost linear red shift of the peaks (corresponding to A_1g_ and E_g_) which can be quantified through Lorentzian fittings. This behavior has been previously observed in other 2D pnictogens, such as LPE and colloidal hexagonal antimonene.^52,53^ The linear dependence of the positions of both modes with temperature (Figure 2c,d) can be attributed to the anharmonic vibrations of the lattice, which involve contributions from lattice thermal expansions to the interatomic potential energy, mediated by phonon–phonon interactions. This behavior illustrates the thermal stability of 2D layered materials, enhancing our understanding of their fundamental properties and potential applications.

**Figure 2:**
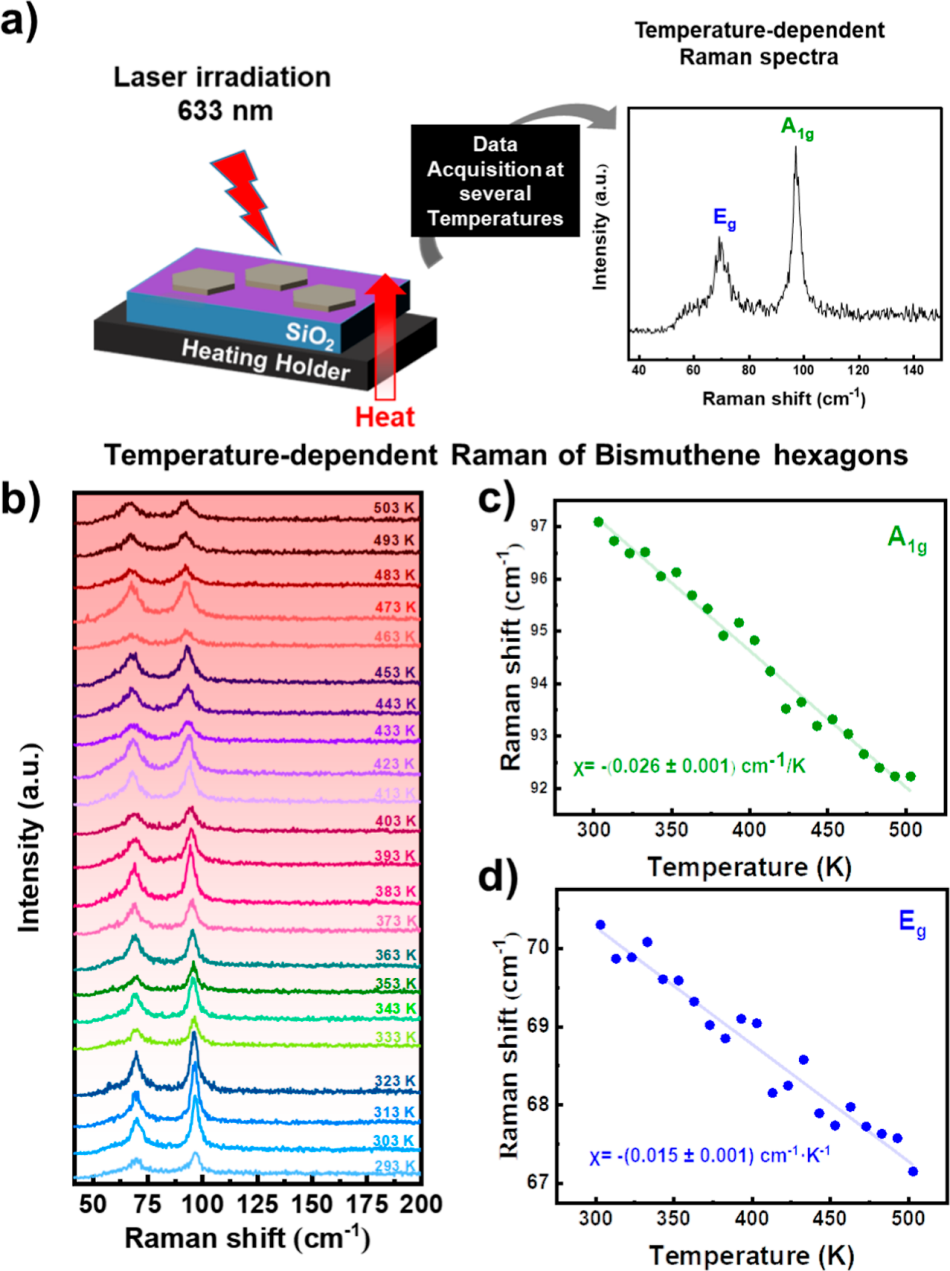
(a) Scheme of the temperature-dependent Raman measurements of hBi on a SiO_2_ wafer. (b) Temperature-dependent Raman spectra of hBi measured at selected temperatures from 293 to 503 K. (c) Temperature dependence of the Raman shift for the A_1g_ mode. (d) Temperature dependence of the Raman shift for the E_g_ mode. Temperature coefficients χ (A_1g_) and χ (E_g_) are extracted from the corresponding linear fit.

The linear dependence on the temperature of both NPs can be observed through the analysis of their Raman spectra, a method commonly referred to as Raman thermometry.^48–51^ The correlation presented in Figure 2c,d follows the established approach consisting of fitting our experimental data using eq 1 ^54^

1 $$\omega \left(T\right)=\omega_{0}+\chi T$$

where ω_0_ is the phonon frequency at 0 K and χ is the first-order temperature coefficient of the corresponding Raman mode (estimated from the slope of fittings in Figure 2b).^55,56^ In the case of hBi, the fitting drives to values of χ (A_1g_) = 0.026 cm^–1^·K^–1^ for the A_1g_ mode and χ (E_g_) = 0.015 cm^–1^ K^–1^ for the E_g_ mode. Moreover, the values that we obtained are comparable with those published for thin Bi films prepared by the physical vapor-phase deposition method.^45^ In terms of the influence of the temperature on the Raman shift, the value for the A_1g_ mode in hBi is similar to that one of Sb nanosheets,^57^ while the value for the E_g_ mode is comparable to monolayer and bilayer graphene,^46^ MoS_2_,^58^ and BP.^59^ Previous research has shown that the first-order temperature coefficient ( χ ) of Raman modes in layered materials is influenced by the van der Waals interlayer interaction. For instance, weak van der Waals interlayer interactions are indicative of smaller χ values.^60–62^ Hence, weak van der Waals interlayer interaction presented in graphene and MoS_2_ leads to small χ values, whereas in Sb and SnSe, the van der Waals interactions are stronger, resulting in a large χ value. On the other hand, the higher the slope, the higher the temperature sensitivity.^46,58^

As the anharmonic coefficients described before are characteristic of the nature of the material, it enables us to calculate the temperature acquired by Bi based NPs during an LHC process. With the aim to evaluate the LHC of hBi and sBiNPs, the power-dependent Raman experiment (Figure 3a) was carried out by irradiation with a focused red laser. In this situation, the temperature of the particles under study can be raised by increasing the power of the excitation laser, without the need of an external heat source. The 633 nm laser was chosen as it allows for more accurate monitoring of the A_1g_ vibrational mode while avoiding the fluorescence that occurs with the 532 nm laser in both hBi and sBiNPs, which manifests around 50 cm^–1^ with higher laser powers (Figures S3a and S4a). The data plotted and shown in Figure 3 are related to both materials for each power energy, in a set of few NPs, using a gradually increasing power from 0.059 to 2.135 mW. Higher powers, such as 2.134 mW, were also tested but were excluded from this study due to the damage caused to the sample by overheating. Both hBi and sBiNSs were measured by means of power-dependent Raman experiments under identical experimental conditions (Figures S3b and S4b). sBiNPs were illuminated by a focused laser beam with gradually increasing power from 0.005 to 2.134 mW. As expected, mode E_g_ and A_1g_ are shifted toward lower energy under high laser-induced heating (Figures S3b and S4b). In addition, in the case of hBi, when the laser power reaches 2.134 mW, a distinct Raman mode was observed around 185 cm^–1^ (Figure S3b). XPS analysis of the hBi has been characterized in our previous publication,^41^ indicating a minimal surface oxidation under inert conditions. This broad new peak can be attributed to α-Bi_2_O_3_, indicating an oxidation produced by the laser irradiation.^63,64^ This band was scarcely found in the laser-power dependent Raman of sBiNPs (Figure S4b), exhibiting less tendency to get oxidized in comparison with hBi employing both laser wavelengths of 532 and 633 nm.

**Figure 3:**
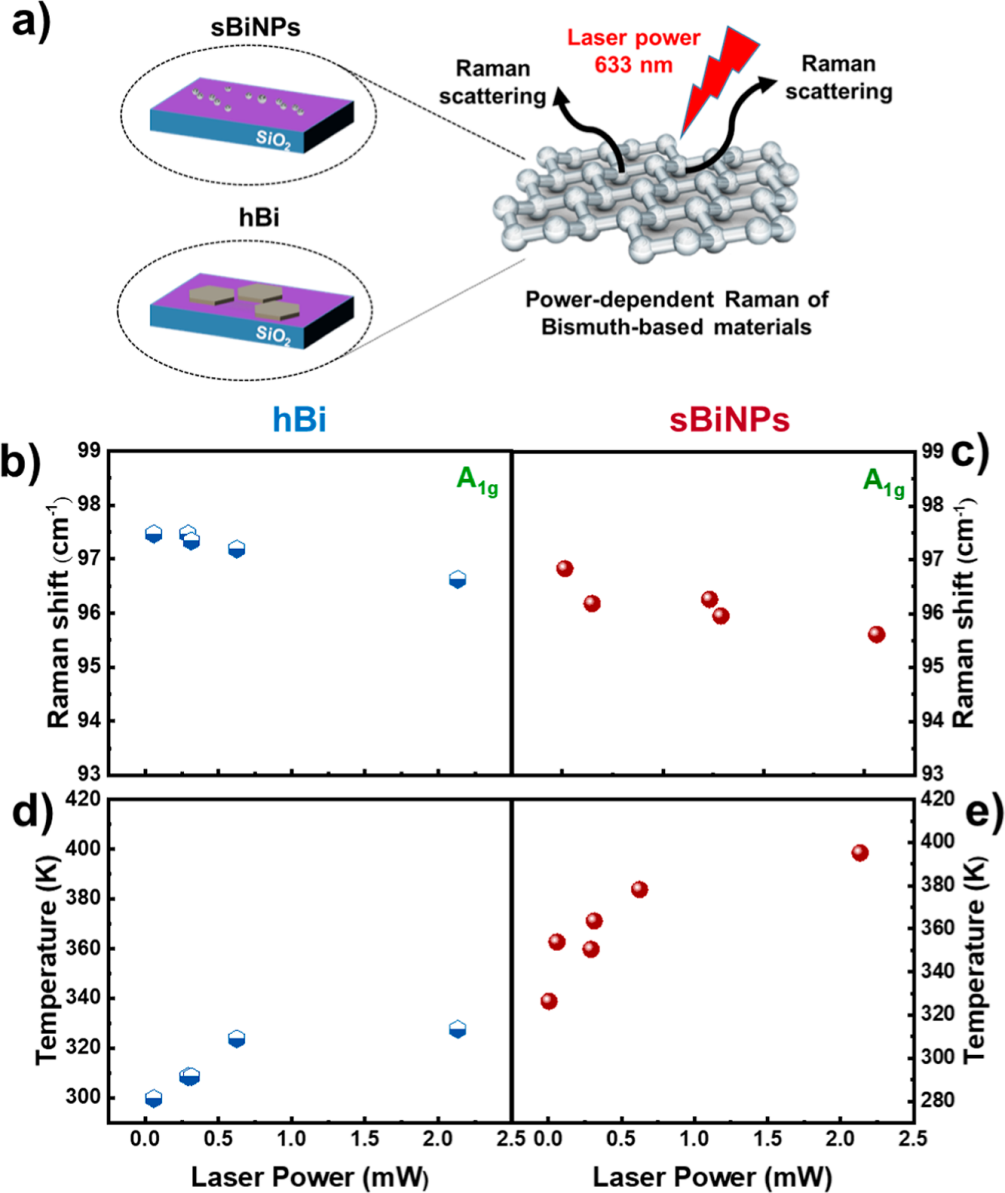
(a) Scheme of the laser-power dependent Raman measurements of hBi and sBiNPs on a SiO_2_ wafer and the laser excitation wavelength. (b) Laser power dependence of the hBi measured at selected laser powers. (c) Laser power dependence of the sBiNPs measured at selected laser powers. (d) Temperature of the hBi as a function of laser power estimated from the corresponding Raman shift. (e) Temperature of the sBiNPs as a function of the laser power estimated from the corresponding Raman shift.

The evaluation of the A_1g_ modes of both hBi and sBiNPs was extracted from the power-dependent Raman measurements. The results obtained for seven NPs of each one are collected in Table S1 of the Supporting Information section. The Raman shift related to the A_1g_ mode was plotted against laser power as shown in Figure 3b,c. Interestingly, both graphs follow a linear dependency when the laser power is below 2.134 mW. However, beyond this threshold, the Raman spectra become irreproducible as peaks broaden because of the decomposition of the material. Since the sensitive response of phonon frequencies in thinner materials allows the performance of optothermal measurements based on Raman spectroscopy, the local heating by laser power excitation of both hBi and sBiNPs are presented in Figure 3d,e, respectively. Using the anharmonic coefficient A_1g_ extracted previously from the temperature-dependent Raman measurements as shown in Figure 2c, we were able to estimate the temperature of the hBi and sBiNPs induced by the increasing power laser.

At first sight, the results presented in Figure 3d,e reveal that using the power of 0.625 mW the temperature that can be achieved in sBiNPs (i.e., 378 K) is ca. 50 K higher than in the case of the hBi (i.e., 327 K). The same outcome was observed for laser powers of 0.315 and 0.293 mW. According to these results, the spherical Bi NPs tend to heat up substantially more than the corresponding hBi. This fact can be a result of the morphology of the nanoparticle, since the efficiency of this process depends on the size and the shape of the nanoparticle.^7,30^ Moreover, the flat hBi NPs possess an elevated heat-dissipation ability due to the contact with the SiO_2_ support.^65,66^ It is well-known that the proper description of the heat conduction from a nano-object to the underlying substrate should consider the thermal contact resistance between them. In the field of 2D materials, this can be accounted for by including “total interface thermal conductance” which is analogous to the “shape factors” commonly used in classical heat conduction models (as described in Figures S14 and S15). In these terms, it is rather intuitive that an increase in the contact area between two objects would lead to a higher shape factor. Conversely, objects with a low surface-to-volume ratio would reduce the shape factor due to the higher volume to area relation (Figure S16). According to this, sBiNPs are expected to achieve higher local temperatures, as their contact with the substrate is limited to a small point, leading to partial heat confinement. In contrast, the low thickness and large contact area of hBi, extending over nearly half of their total surface, facilitated efficient heat dissipation to the substrate (Figure S17).

When compared to a similar 2D system on a Si/SiO_2_ wafer, such as microexfoliated and colloidally synthesized antimonene, the hBi samples reach higher temperatures with lower power irradiations.^52,57^ To reach a temperature of 300 K in antimonene, a power of 4 mW with a 532 nm laser is required. In contrast, hBi can achieve the same temperature by using only half that power with a 633 nm laser (Figure 3d).

Since the size and the morphological effects have an important influence on the LHC process, we contrast the Raman thermometry properties of sBiNPs of diameters of 55 ± 10 nm (Figure S6a,b), by comparison with functionalized spherical Au NPs with 15 ± 5 nm (Figures S6c,d, S7 and S8). Although the sBiNPs exhibit size dispersity, the higher resistive losses of bismuth result in a broader extinction peak, which prevents significant changes in the position of the absorption cross section spectrum. Due to the lack of Raman signal, Au NPs were functionalized with the organic molecule 4-methylbenzenethiol (PMBT) to monitor the temperature evolution (Figure S9). This functionalization enhances the Raman signal of Au NPs, as has been previously published in reports about Au NPs and Ag films.^67,68^ The obtained results are presented in Table S1. The comparison of the temperature raised at different laser powers is shown in Figure 4. In particular, the Raman shift by AuPMBT NPs during the temperature-dependent Raman experiment (Figure S9) is monitored by following the peak at 1079 cm^–1^ from 298 to 473 K. The peak at 1079 cm^–1^ (298 K) of the molecule PMBT was selected to monitor the shift because of its great intensity in comparison with the other peaks. The power-dependent Raman measurements of the AuPMBT NPs (Figure S10) were also carried out using the same laser settings as before, employing a gradually increasing laser power from 0.005 to 2.134 mW. Beyond that power, the signal of AuPMBT NPs that allows us to follow the experiment is not detectable since the covalent functionalization does not remain after 200 °C.

**Figure 4:**
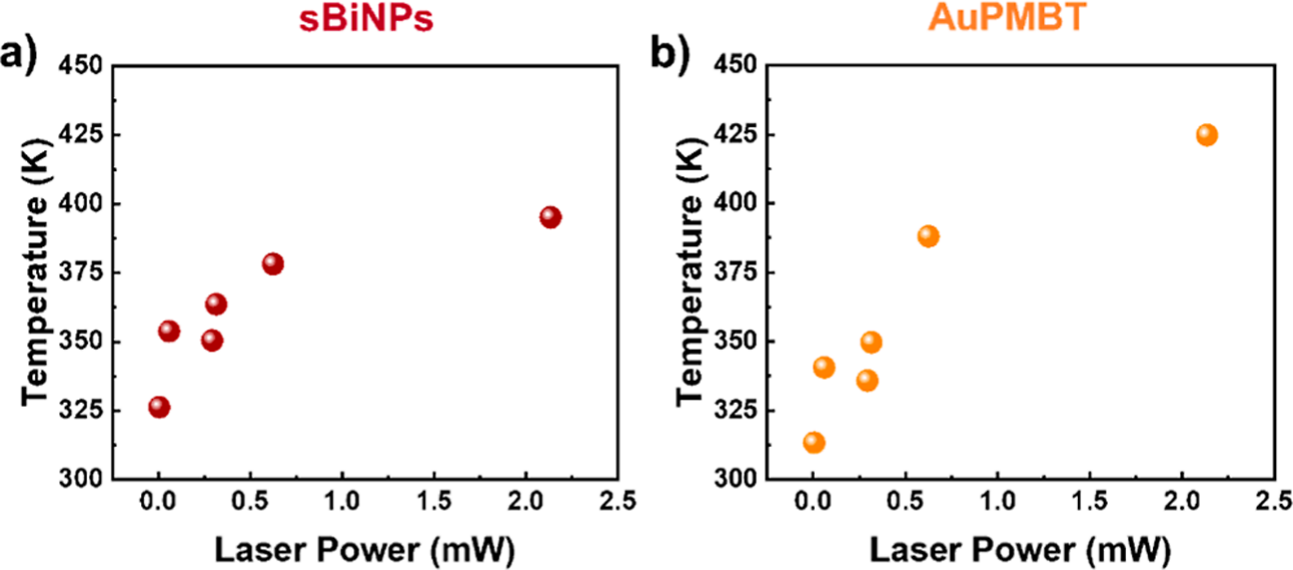
(a) Temperature of the sBiNPs as a function of laser power applied. (b) Temperature of the AuPMBT NPs as a function of the laser power applied.

The energy of the monitored modes was extracted from the power-dependent Raman spectra presented in Figure S10a, which was then plotted versus the laser power in Figure S10b. Finally, Figure 4a,b compares the local heating by laser power excitation for both sBiNPs and AuPMBT NPs, respectively. From that comparison (Figure 4), we observe that a temperature of over 400 K can be achieved in both materials using an excitation power of 2.134 mW. Similar behavior for both NPs can be observed also at lower laser powers, such as 0.315 and 0.293 mW. The data plotted in Figure 4 highlight that sBiNPs exhibit significant heating when irradiated with a laser. The fact that the spherical Bi NPs present a similar performance to the spherical Au NPs points out Bi as an efficient photothermal system. Moreover, due to the synthetic versatility of the colloidal synthesis employed to obtain the Bi NPs, we have been able to produce sBiNPs with diameters ranging from 140 ± 40 nm to demonstrate that larger spherical morphologies enable more efficient heat transfer compared to hexagonal nanoparticles (Figure S11). The morphological characterization was completed with DLS measurements, showing in Figure S11c the size and number of aggregates. The temperature reached by these NPs has also been measured using Raman thermometry, employing powers ranging from 0.005 to 0.625 mW. The results are presented in Figure S12 and reveal that using the power of 0.625 mW the temperature that can be achieved in sBiNPs with approximately 140 nm in size (i.e., 400 K) is still higher than in the case of hBi (Figure 3d). The data are similar to the sBiNP of approximately 55 nm diameter but with a considerably lower standard error of the median in the data of Table S1 in the Supporting Information section.

## 4 Discussion

In view of the experimental section above, we can conclude that both materials, spherical Bi and Au NPs, have comparable LHC capabilities. To support the experimental results, numerical calculations corresponding to the energy and line width of the localized surface plasmon resonance (LSPR) for spherical Au and Bi NPs with different diameters (*D*) were accomplished (Figure 5). The data were extracted from the extinction spectra calculated using the Mie Theory and double-checked with previous results in the literature.

**Figure 5:**
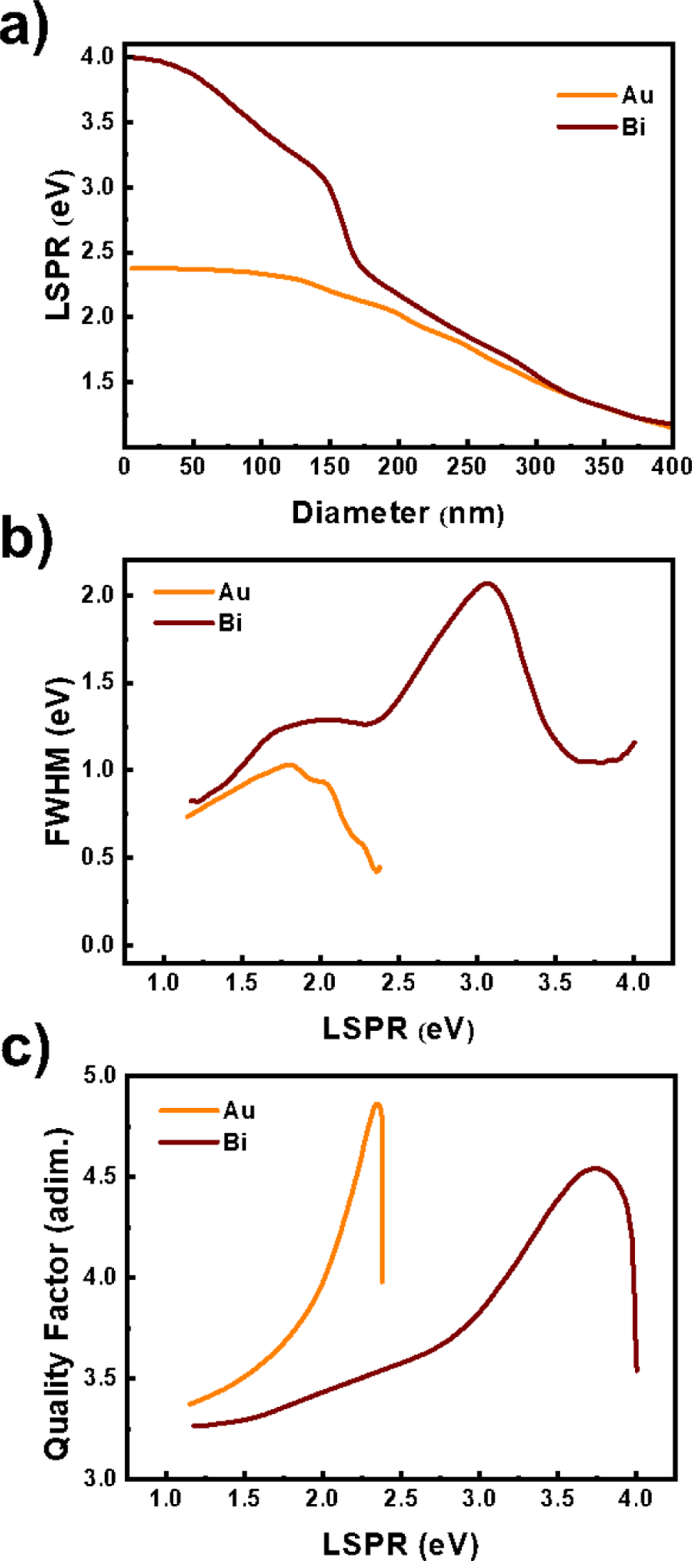
Optical properties of spherical Bi and Au NPs. The data in the plots have been extracted from extinction spectra calculated by means of the Mie Theory. (a) Represents the position of the LSPR as a function of the NP diameter. (b) Represents the fwhm of the LSPR observed at different energies, thus for NPs of different diameter. (c) Presentation of the corresponding quality factor.

It is well-known that the LSPR effect of small plasmonic NPs strongly depends on the electron density. According to the previously published work, as the diameter increases, the LSPR is red-shifted. Furthermore, the depolarization effects tend to appear for larger NPs (*D* > 200 nm), leading to an independent LSPR response which explains the proximity of the curves in Figure 5 above this size. However, for smaller particles, the size dependence occurs differently for spherical Au and Bi NPs. In the case of the smallest Au NPs (orange line in Figure 5a), the size-dependent LSPR response consists of an asymptotic increase of the LSPR energy up to 2.4 eV. For the smallest Bi NPs (red line in Figure 5a), the red shift increases up to 4 eV. This larger tuning of Bi NPs can be considered as an advantage versus Au for certain applications such as photocatalysis.

Figure 5b represents the line widths (full width at half-maximum, fwhm) observed in Figure 5a, in front of the LSPR energy of both materials. The threshold for the interband transition from the 5d orbitals is estimated at around 2.3 eV and the narrowest line widths observed for small Au NPs are over <0.4 eV. The broadening is attributed to radiative damping, which depends on the electron density and the size of the NP. For this reason, the broadening increases up to the point where the depolarization effects affect the radiative rate, which occurs for diameters around 200 nm. The situation is rather different for Bi NPs since Bi exhibits a complex electron structure that allows interband transitions even for very low energies,^69,70^ for example, at 1.5 and 2.5 eV. Within this range, the fwhm of Bi is twice the one observed for Au NPs on average. Moreover, the broadening presented in Bi is reduced for smaller NPs, supporting LSPRs at higher energies. As a result, the quality factors (*Q* = *E*_LSPR_/Δ*E*_LSPR_, represented in Figure 5c) exhibited by Bi NPs in the range between 3 and 4 eV are comparable to the best figures observed for Au. Indeed, the real part of the dielectric function of Bi is negative, and the imaginary part is very low in this range (Figure S13), highlighting again the potential of Bi for plasmonic applications at high energies.

However, we should distinguish between those applications relying on an increase in the local density of optical states, such as light enhancement, SERS, or sensing, and those applications requiring high resistive loss, such as photocatalysis, thermoplasmonics, or photothermal therapy. On the one hand, narrow line widths and huge near-field enhancement are crucial for applications based on the increase of the local density. In this field, Au is unbeatable in the visible range (from 2.4–1.7 eV). According to the quality factors predicted in Figure 5c, Bi could be a worthy alternative to perform this kind of application in the UV region. On the other hand, in the case of applications that require a resistive loss, the difference with Au is not that clear. The cross section absorption spectra of spherical Au and Bi NPs of several diameters are presented in Figure 6a. The behavior is rather similar between Au NPs of several diameters, with a clear absorption maximum that corresponds to the LSPR (around 2.3 eV). In contrast, the cross section absorption spectra of Bi NPs present other features related to the above-mentioned complex and interband transitions. This means that Bi NPs exhibit a very efficient absorption, even out of the resonance. In addition, a high absorption cross section in the visible region is estimated for LSPR of Bi NPs within the range of 50–150 nm of diameter, despite the LSPR effects occurring at 3 to 3.7 eV. Furthermore, BiNPs have great potential for photothermal therapy as already shown.^28,29^ Even so, they need to be capped to avoid their oxidation upon irradiation in aqueous media like the biological environments and improve their ability to generate local heat (Table S2). Nevertheless, this difference of stability with the AuNPs can be of great advantage, as the controlled light-induced oxidation of the Bi can produce toxic Bi oxides species that have synergistic therapeutic effects with the photothermal therapy.^27^

**Figure 6:**
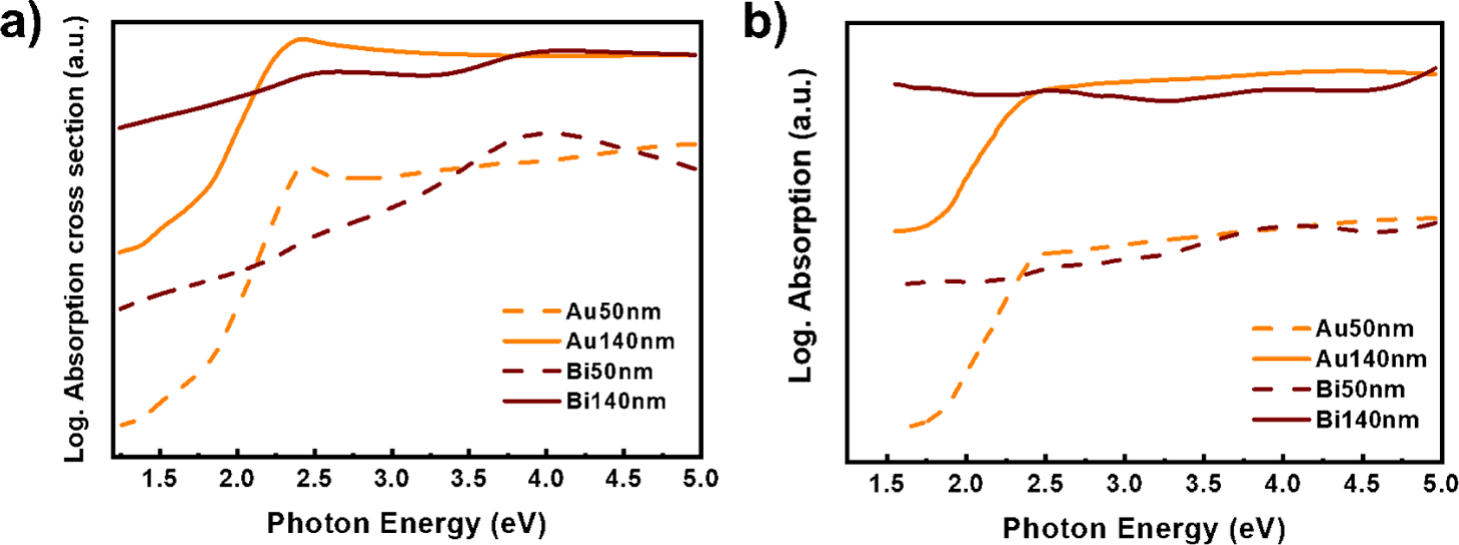
(a) Absorption cross section spectra of spherical Au and Bi NPs with diameters of 50 and 140 nm. (b) Effective absorption of Au and Bi NPs. Despite the red shift related to the size increase, the differences in the absorption efficiency are scalable to the NP diameter.

Figure 6b compares the effective absorption, which is the amount of light converted to heat for both Bi and Au NPs. While the absorption of Au is quite efficient above 2 eV, Bi NPs present a prominent absorption in the UV, visible, and NIR regions (Figure S18). It is important to note that, in certain applications such as photocatalysis, this magnitude should consider only the absorption close to the interface (mean free path, MFP), since the electron generated cannot reach the surface in larger NPs. The low population in the conduction band of Bi allows the conduction electrons to move along micrometric distances. In the case of Au NPs, MFP is on the order of tens of nanometers, ca. 37 nm, so part of the carriers photogenerated could not be employed.

According to the results commented above, smaller Au and Bi NPs present a LSPR of 2.4 and 4 eV, respectively. Bi NPs evaluated in this work (with *D* > 200 nm) exhibit a lower LSPR effect; therefore, the heat generated is not produced because of the LSPR effect (Figure 5a). However, the fwhm (Figure 5b) and the absorption cross section spectra (Figure 6a,b) explain why both Bi and Au performances in Figure 4 are comparable.

## 5 Conclusions

The LHC efficiency of hBi was experimentally investigated on a SiO_2_/Si substrate. First, the linear temperature dependence of the Raman mode positions revealed the first-order temperature coefficients of χ (A_1g_) = −0.026 and χ (E_g_) = −0.015 cm^–1^ K^–1^, for the A_1g_ and E_g_ mode, respectively. Using these coefficients, the local temperature increase under different excitation powers is determined. The efficiency of these is benchmarked using sBiNPs and functionalized Au NPs, namely AuPMBT. It was observed that AuPMBT exhibits a slightly higher LHC reaching up to 425 K under 2.1 mW of excitation at 633 nm. In the case of sBiNPs, the temperature was increased up to 400 K under the same excitation conditions, while a temperature 325 K around the hBi was reached. This superior light-to-heat conversion efficiency of sBiNPs arises from their morphology, which enhances thermal confinement at the nanoparticle–substrate interface, rather than from intrinsic material properties. As the spherical Au and Bi NPs have shown comparable LHC, we should attribute the lower thermal contrast found in hBi to a higher diffusion to the substrate. Finally, a set of numerical simulations is provided to compare the light absorption in spherical Au and Bi NPs. These findings are essential for the development of further applications of Bi in photothermal and thermoplasmonic applications.

## Supplementary Material



## Notes

Experimental details, DLS data, Raman spectra, SEM and TEM images, histograms of particle size distributions, temperature- and power-dependent Raman measurements, dielectric function comparison, and summary table of spectral data (PDF)
