# Supplementary material for: Hexagonal Bismuthene and Bismuth Nanoparticles for Light-To-Heat Conversion
Source: ACS Appl Mater Interfaces. 2025 Aug 28;17(36):51457–67. doi: 10.1021/acsami.5c11661 (PMC12442010; doi:10.1021/acsami.5c11661)
Supplement: Supplementary file 1 [file am5c11661_si_001.pdf]

Supporting Information

Hexagonal bismuthene and bismuth nanoparticles for light-to-heat conversion.

Marta Alcaraz, Pau Congost-Escoin, Christian Dolle, Josep Canet-Ferrer, Gonzalo Abellán**

Instituto de Ciencia Molecular (ICMol),Universidad de Valencia, 46980 Paterna, Spain

Emails of corresponding authors: jose.canet-ferrer@uv.es ; gonzalo.abellan@uv.es

CHARACTERIZATION TECHNIQUES

Substrate preparation: Si/SiO_2 surfaces were washed and sonicated for 10 minutes with basic piranha (x3) and acetone (x1). Subsequently, the cleaned substrates are dried under N2 flow.

Transmission Electron Microscopy (TEM): Images were obtained in a JEOL JEM 2100 FX TEM system with an accelerating voltage of 100 kV. The microscope has a multiscan charge-coupled device (CCD) camera ORIUS SC1000 and an OXFORD INCA X-Ray Energy Dispersive Spectroscopy microanalysis system. For the preparation of TEM samples, the materials were dispersed in $CHCl_3$ and deposited on lacey formvar/carbon copper grids (300 mesh).

Scanning electron microscopy (SEM): the SEM images were acquired in a SCIOS 2 field emission scanning electron microscope, equipped with an electron column, offers a beam resolution of up to 0.7 nm at 30 kV in STEM mode and 1.2 nm at 1 kV with deceleration. Its beam current ranges from 1 pA to 400 nA, with an acceleration voltage adjustable from 200 V to 30 kV, providing high precision and versatility for advanced analyses.

Dynamic light scattering (DLS): DLS measurements were collected in a Zetasizer Ultra Malvern Panalytical. The algorithm used for acquired data was Adaptative Correlation. The samples were sonicated in a sonication bath for 15 min before measurements at room temperature. sBNPs tend to aggregate in solution driving an estimated average size-distribution. For this reason, their average size-distribution of sBNPs are estimated by means of counting statistics from TEM images.

Raman spectroscopy: Raman spectroscopy characterization was carried you using a Horiba LabRam HR Evolution spectrometer, employing a HeNe laser (633 nm, power laser 0.01%–100%). Temperature and power-dependent Raman spectra were acquired using a 50x objective and 100x objective, respectively with spot sizes of 1.55 μm and 0.86 μm for each one. An EMCCD camera was employed to collect the backscattered light dispersed by 600 grooves per mm grating providing a spectral resolution of 0.4 cm^{-1}. Temperature-dependent Raman measurements were performed in a THMS 600 temperature-controlled stage from Linkam Scientific Instrument with a constant flow of nitrogen to stabilize the temperature. The spectra were acquired from 293 K to 503 K through 10 $K\cdot min^{-1}$ steps. The resulting laser-dependent Raman spectra are the average of seven nanoparticles. The corresponding Raman spectra were then constructed by processing the data using Lab Spec 6 software.

ADDITIONAL EXPERIMENTAL DATA

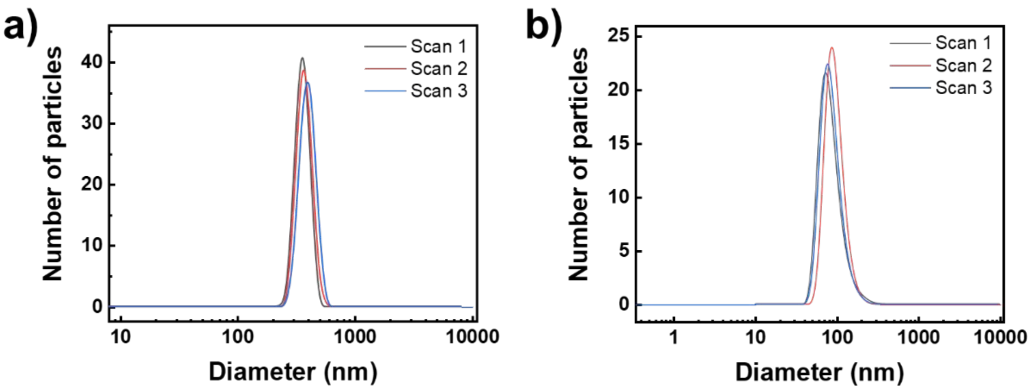

Figure S1. a) DLS measurements of the particle size of hBi. **b)** DLS measurements for the particle size of sBiNPs. Both represented versus number of particles.

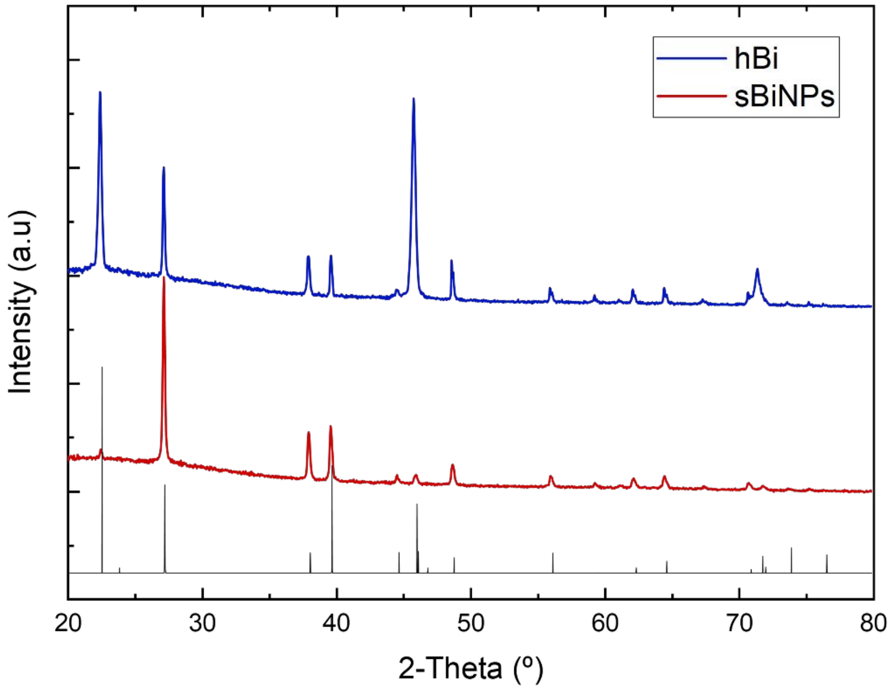

Figure S2. Surface XRD of the synthesized Bi NPs.

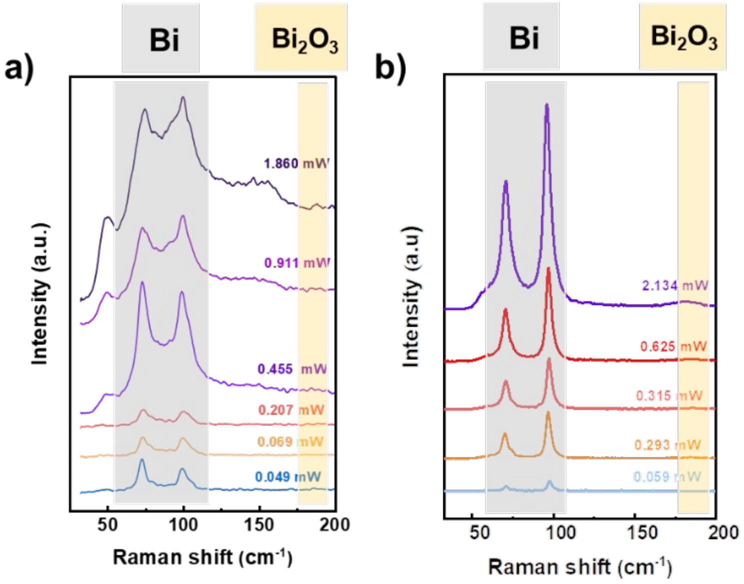

Figure S3. Raman spectra of hBi using **a)** laser 532 nm and **b)** 633 nm at different laser powers.

During the irradiation with 532 nm laser both hBi and sBiNPs are overheated, and the process can not be monitored effectively.

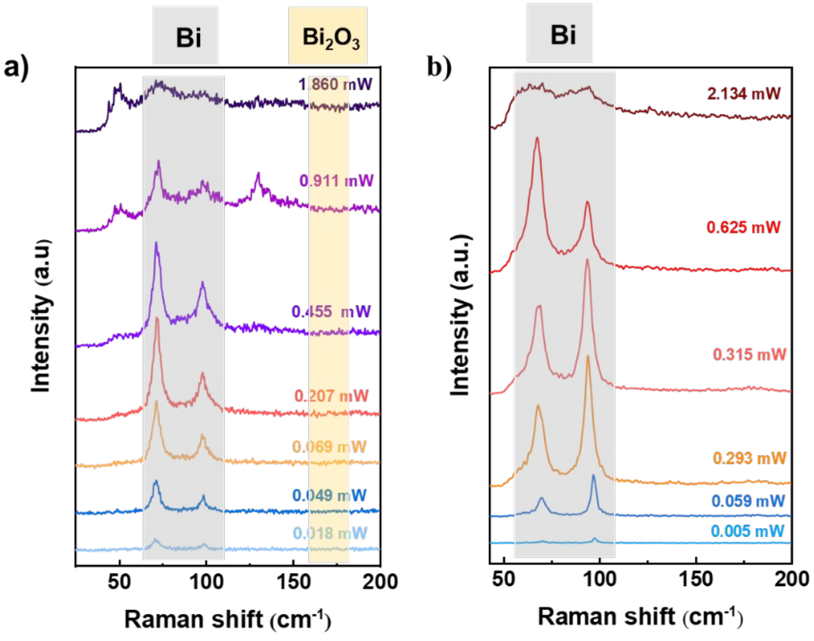

Figure S4. Raman spectra of sBiNPs using **a)** laser 532 nm and **b)** 633 nm at different laser powers.

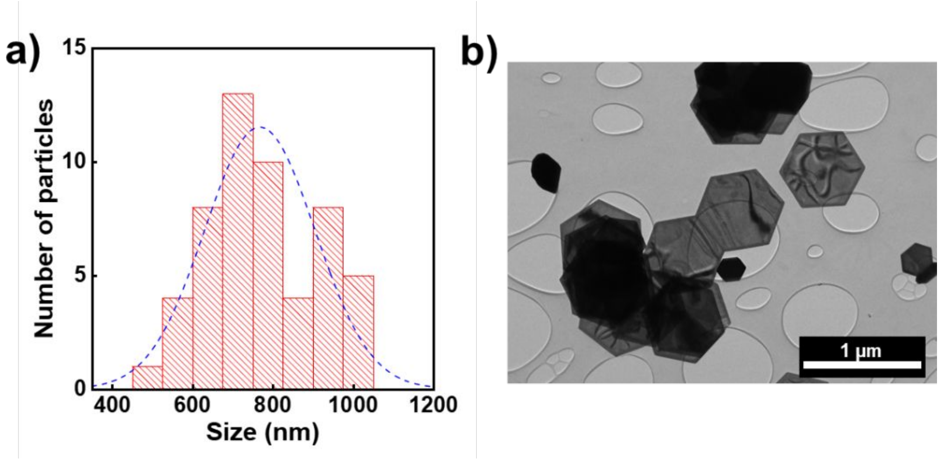

Figure S5. a) Histogram of lateral size hexagonal bismuthene (hBi) nanosheets obtained from TEM images. Measurements were performed on more than 60 individual flakes, and the lateral dimension was defined as the longest axis of each hexagon. Average size of

760 ± 140 nm. **b)** Representative TEM image showing the hexagonal morphology and lateral dimension of hBi flakes deposited on a lacey carbon grid.

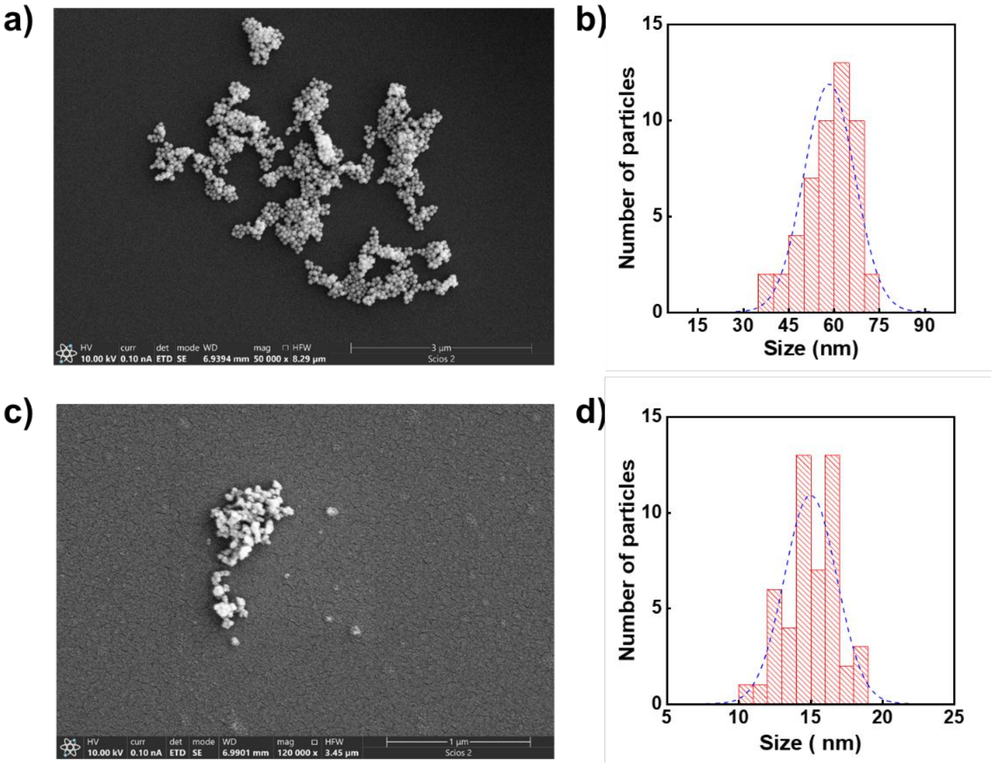

Figure S6. a-b) SEM image of sBiNPs (a) and histogram sizes (b). **c-d)** SEM image of AuPMBT NPs (c) and histogram sizes (d). A total of 50 particles were analyzed.

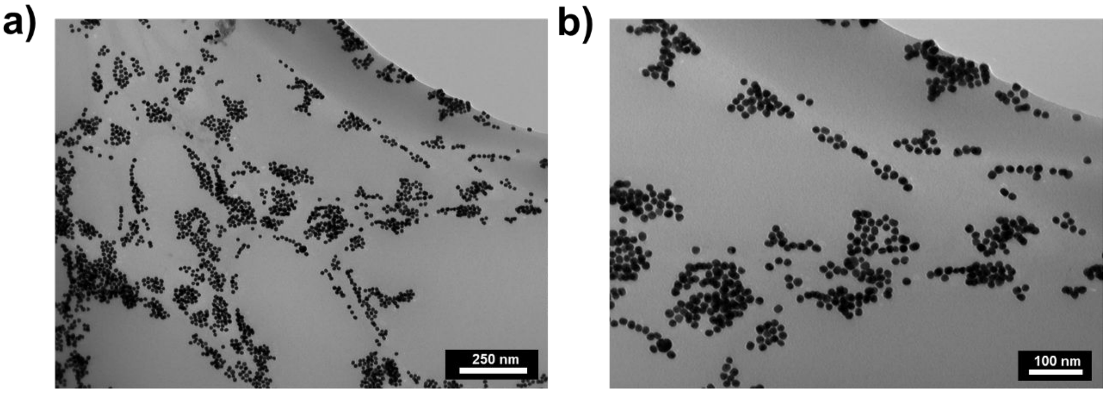

Figure S7. a-b) TEM images of AuPMBT NPs.

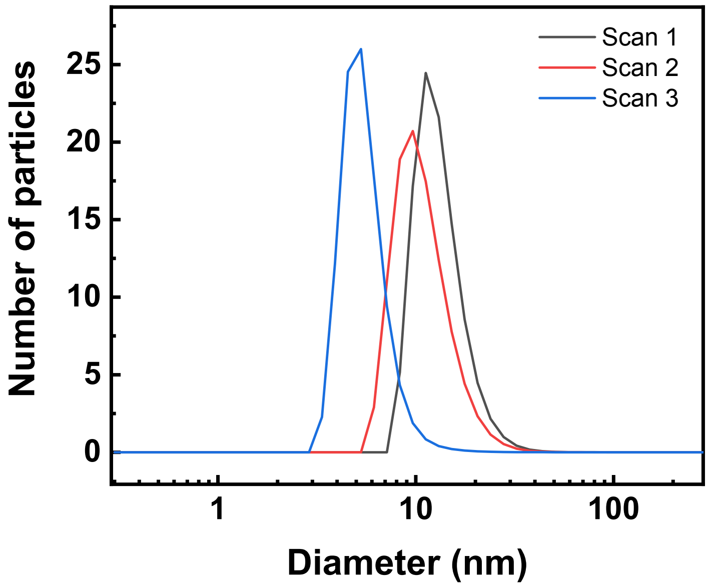

Figure S8. DLS measurements for the particle size of AuPMBT NPs represented versus number of particles.

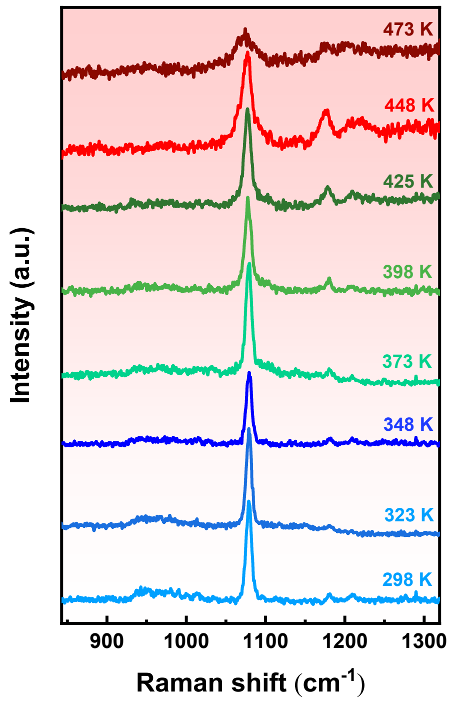

Figure S9. Temperature-dependent Raman spectra of AuPMBT nanoparticles.

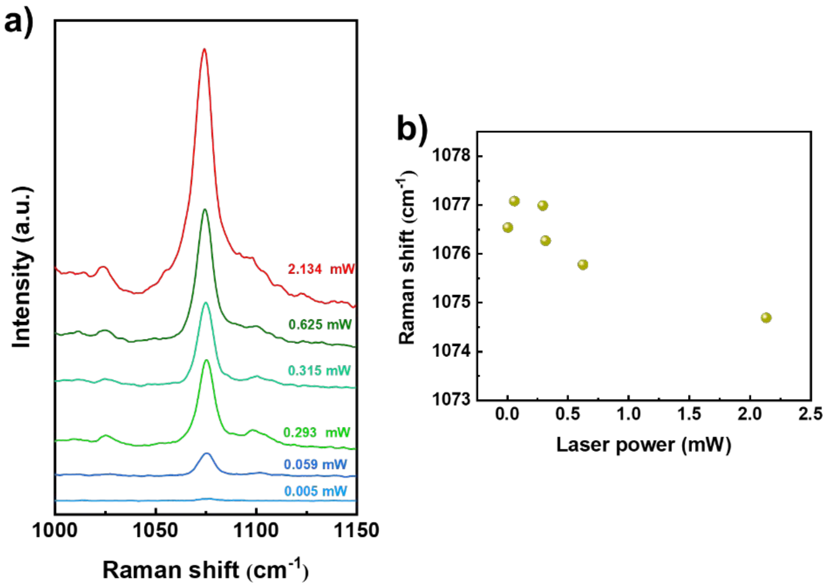

Figure S10 a) Laser-power dependent Raman spectra of AuPMBT nanoparticles. **b)** Laser power dependence of AuPMBT nanoparticles measured at selected laser powers.

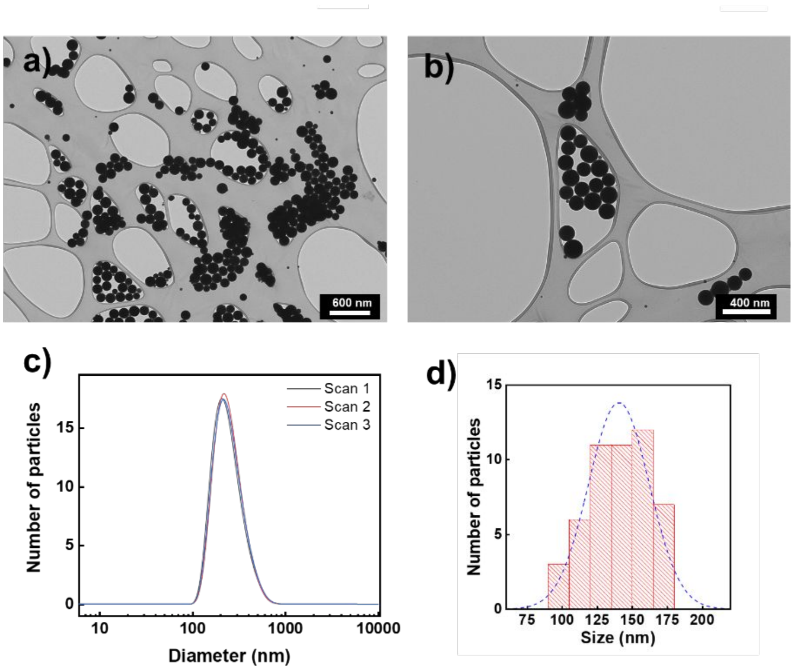

Figure S11. a-b) TEM images of sBiNPs with a diameter in the range of 140 ± 40 nm. **c)** DLS measurements for the sBiNP represented versus number of particles. **d)** Histogram of 50 NPs.

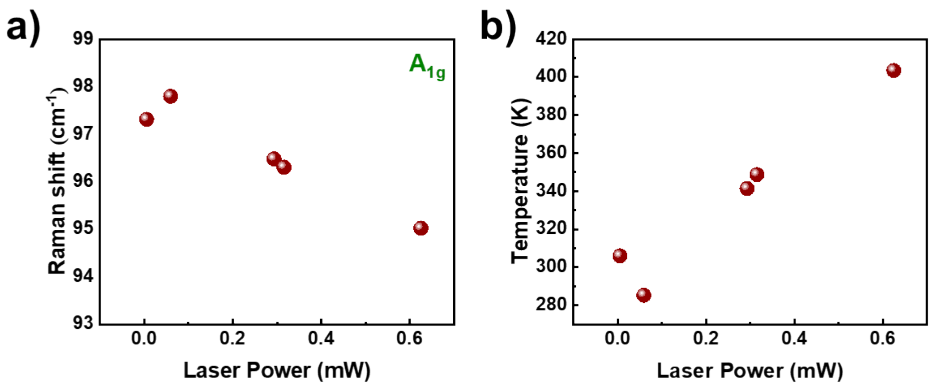

Figure S12. a) Laser power dependence of the sBiNPs of 140 ± 40 nm measured at selected laser powers. **b)** Temperature of the sBiNPs of 140 ± 40 nm as a function of the laser power estimated from the corresponding Raman shift.

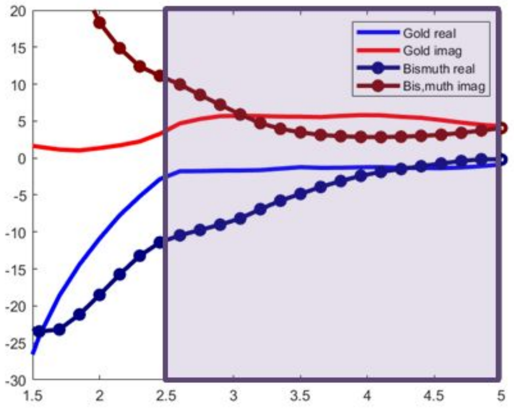

Figure S13. Comparison of dielectric functions of gold and bismuth materials.

The data presented in Table S1, for both temperature-dependent and power-dependent Raman measurements, are the result of the average of seven NPs. We provide the Standard Error of the Median (SEM) as a measure of dispersion in these data. The power-

dependent Raman experiments were conducted on hBi, sBiNPs of 55 ± 10 nm, sBiNPs of 140 ± 40 nm, and AuPMBT NPs, allowing us to assess the impact of laser power on the anharmonic A_{1g} coefficient shift. Similarly, temperature-dependent Raman experiments were performed on AuPMBT NPs to examine variations in the anharmonic coefficient shift as a function of temperature. These results help us evaluate the phonon behavior in the different nanostructures, providing insights into their thermal and optical stability under varying conditions.

**Power-dependent Raman experiments of hBi**		
Laser Power (mW)	Mean of Shift of the Anharmonic A_{1g} coefficient (cm^{-1})	Standard Error of the Median (SEM)
0.059	97.466	0.295
0.293	97.463	0.340
0.315	97.334	0.691
0.625	97.184	0.158
2.134	96.623	0.802
**Power-dependent Raman experiments of sBiNPs of 55 ± 10 nm**		
Laser Power (mW)	Mean of Shift of the Anharmonic A_{1g} coefficient (cm^{-1})	Standard Error of the Median (SEM)
0.005	96.831	0.195
0.059	96.185	0.333
0.293	96.263	0.341
0.315	95.954	0.423
0.625	95.612	0.615
2.134	95.215	0.890
**Power-dependent Raman experiments of sBiNPs of 140 ± 40 nm**		
Laser Power (mW)	Mean of Shift of the Anharmonic A_{1g} coefficient (cm^{-1})	Standard Error of the Median (SEM)
0.005	97.310	0.882
0.059	97.797	0.681
0.293	96.477	0.734
0.315	96.304	1.187
0.625	95.019	1.444
**Temperature-dependent Raman of AuPMBT NPs**		
Temperature (K)	Mean of Shift of the Anharmonic coefficient (cm^{-1})	Standard Error of the Median (SEM)
298	1076.994	0.658

323	1076.922	0.587
348	1076.336	0.106
373	1075.840	0.646
398	1075.792	0.491
448	1073.960	3.043
**Power-dependent Raman of AuPMBT NPs**		
Laser Power (mW)	Mean of Shift of the Anharmonic coefficient (cm^{-1})	Standard Error of the Median (SEM)
0.005	1076.540	0.261
0.059	1077.084	0.753
0.293	1076.989	0.669
0.315	1076.270	0.882
0.625	1075.781	0.564
2.134	1074.689	1.313

Table S1. Temperature and power-dependent Raman data from the spectra of sBiNPs, hBi and AuPMBT NPs.

NPs morphology influence in thermal diffusion effect.

During the discussion we attribute the lower temperature achieved in hBi to the higher thermal diffusion of this low aspect ratio NPs, and this argument is further reinforced by geometrical considerations. Let’s begin with a simple comparison of heat diffusion between a spherical NP and a disk-like NP. The spherical NP can be considered as a point-like heat source while the nanodisk can be considered as a wide contact area heat source. Indeed, our hBi flakes, with diameters close to one micron and height in the order of ten nanometers, have almost half of its effective area in contact with the substrate. Considering both NPs as heat engines with a rate of $1mW\cdot s^{-1}\cdot \mu m^{-3}$, we can observe a clearly larger temperature contrast with respect to the substrate in the case of spherical NPs. The contrast is even higher considering a constant heating power of 1mW (non-normalized) for both.

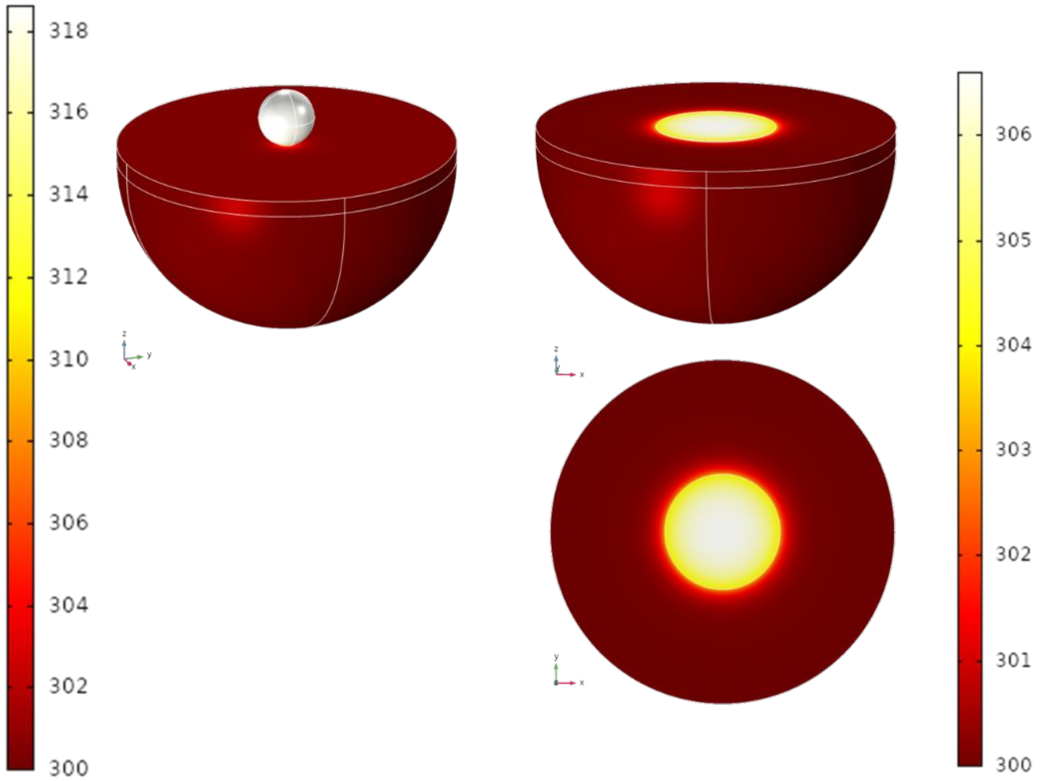

Figure S14. Temperature contrast between spherical and disk-like geometries under similar heating conditions. The sphere exhibits a high aspect ratio, with its contact with the substrate limited to a single point, while the disk shows a low aspect ratio, with almost half of its effective surface in contact with the substrate.

Notice that the above simulations are carried out taking into account the volume of the NPs. However, for the sake of a simpler explanation instead of comparing disks to spheres we propose a comparison between partially embedded spheres. All the embedded spheres (of a given diameter) are assumed to exhibit the same heating power. First, we compare spherical NPs of 500 and 50 nm of diameter (see Figure S14). In both simulations, the contact between the sphere and the substrate is minimum.

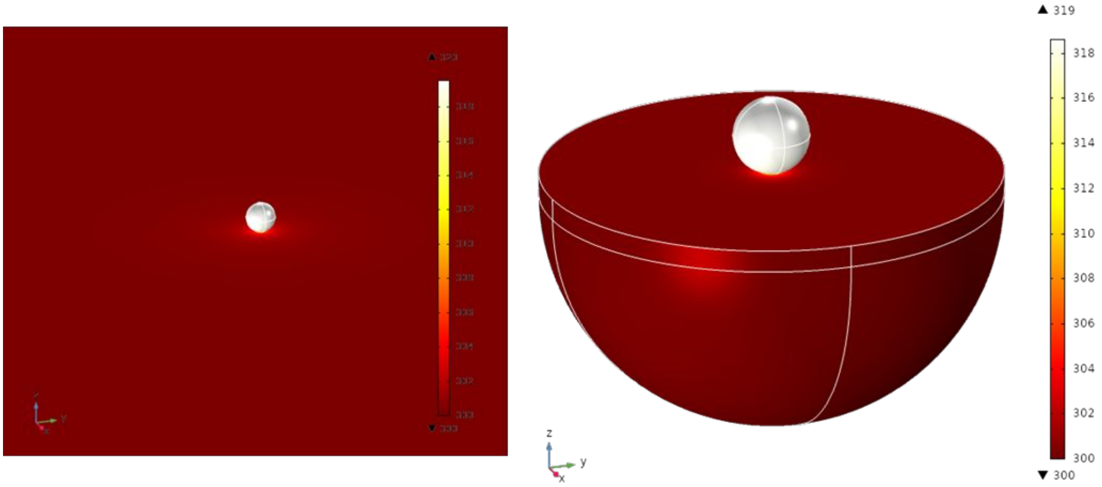

Figure S15. Temperature maps for spheres of 50 nm (left) and 500 nm (right) generating a heating rate of 1 mW·s^{-1}·μm^{-3}.

Then, in Figure S15 we plot again the same spherical NPs with a diameter of 500 nm in two different situations. In Figure S16 a, the sphere is gently touching the surface so it can consider a point-like heat source, while in Figure S16 b, half of the sphere is embedded into the substrate, thus increasing the thermal contact. In the second case the temperature contrast is below 1K.

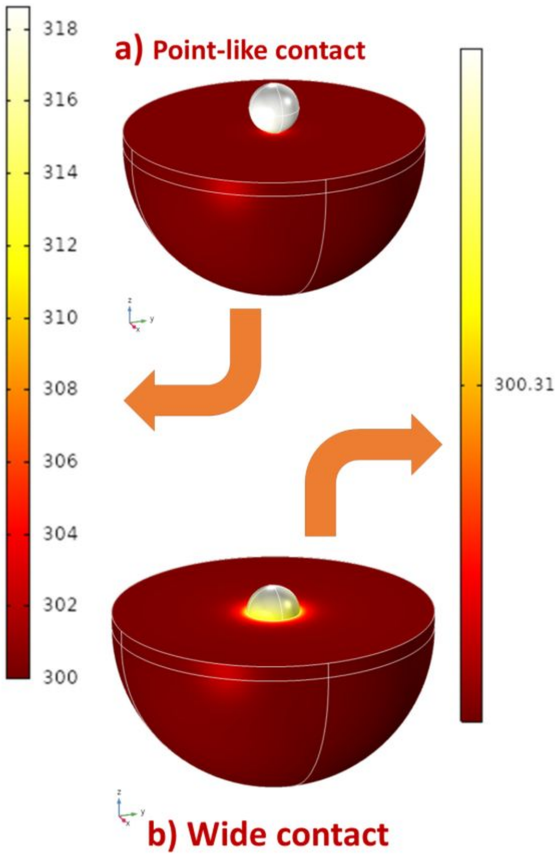

Figure S16. Simulated temperature distribution under identical heating conditions comparing a) Point-like contact and b) Wide contact geometries. The upper configuration (a) represents a spherical nanoparticle with contact limited to a single point, whereas the lower configuration (b) illustrates a disk-like geometry with an extended contact area. The color bars and arrows indicate the temperature scales corresponding to each system. The spheres show a higher local temperature due to reduced heat dissipation into the substrate.

By reproducing these simulations for spheres of 50 and 500 nm with different degrees of embedding we can obtain the plot of Figure S17. These represent the temperature of both kind of spheres depending on the embedding degree. The data are plotted as a function of the penetration depth, of the embedded cross-sectional area, and of their corresponding normalized values. It is normalized to the sphere radius in the case of the penetration depth and on the sphere surface in the case of the embedded section. The results are quite

clear, despite some quantitative differences that we attribute to normalization we can conclude that the increase of the section tends to reduce the maximum temperature that a NP can achieve.

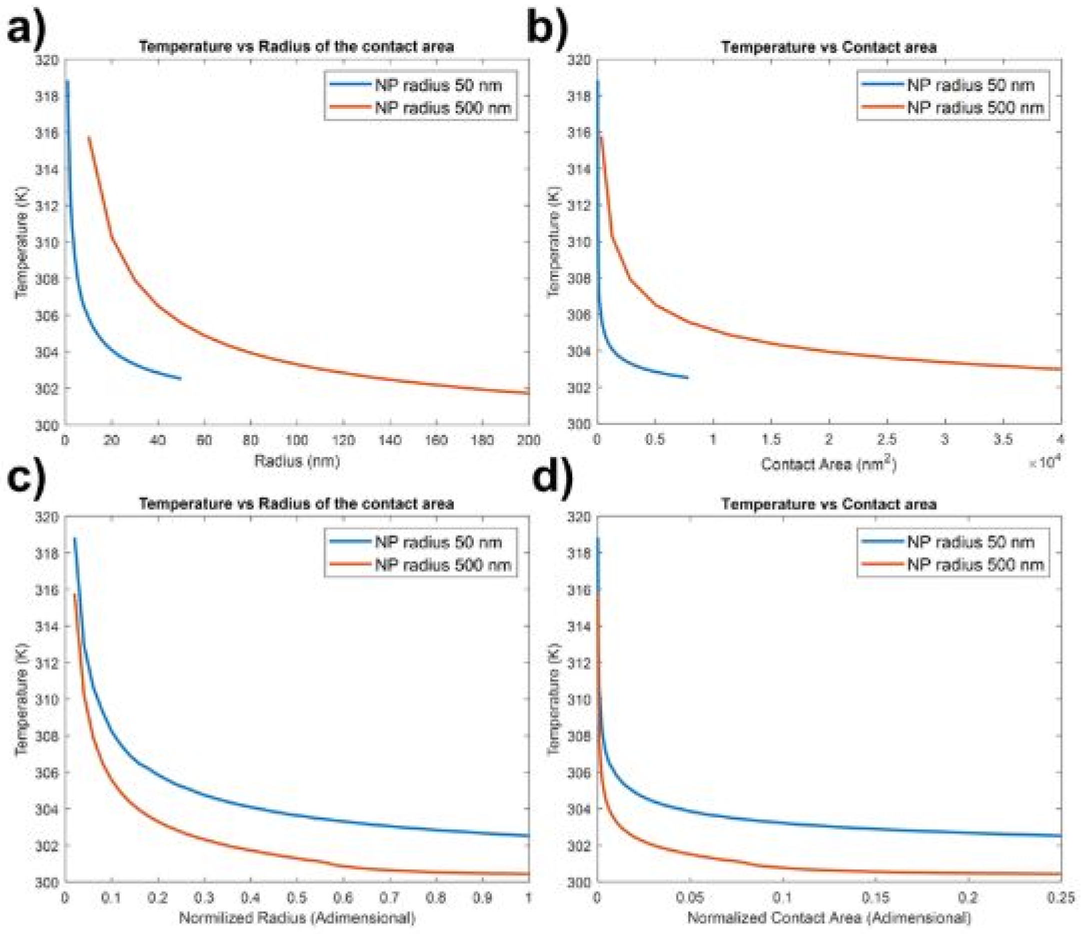

Figure S17. Temperature profiles as a function of contact area for spherical nanoparticles of different sizes (NP radius 50 nm and 500 nm) partially embedded in the substrate. Each panel shows the steady-state temperature reached under identical heating conditions for increasing contact extent versus a) contact radius, b) contact area, c) normalized contact radius and d) normalized contact area. In all cases, smaller contact areas (lower embedding degrees) lead to higher local temperatures due to reduced thermal dissipation into the substrate.

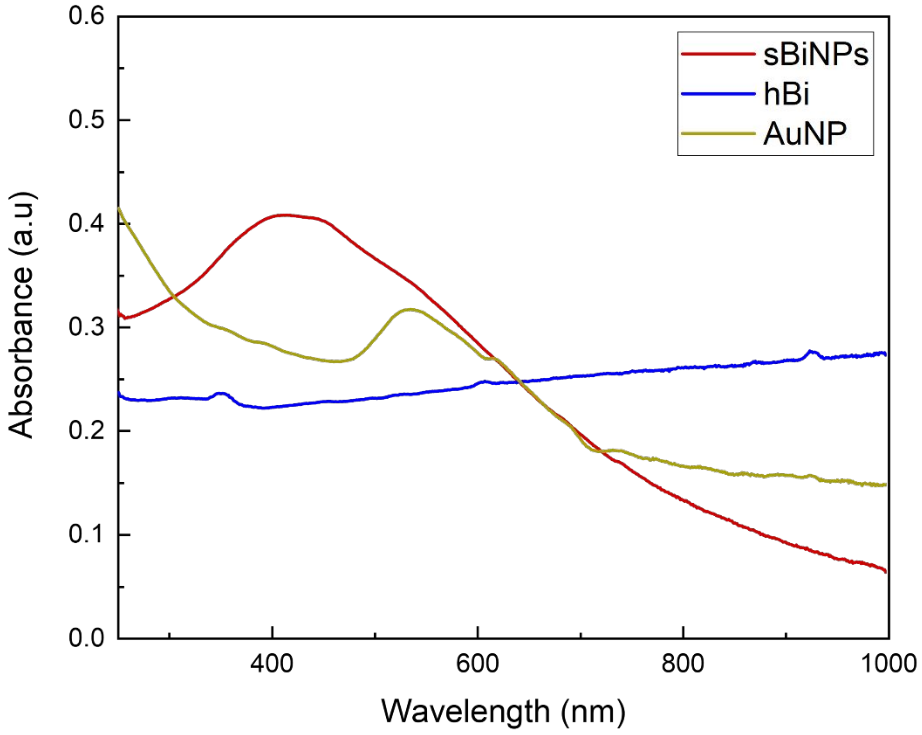

Figure S18. UV-vis spectra of the hBi, sBiNPs and AuNPs.

Material	PTCE	Description	Intrinsic Toxicity	References
**Bi NP**	29.1 %	Bismuth-dodecanethiol nanoparticles capped with poly(N-vinyl-2-pyrrolidone)	Non-toxic	1
**Bi NP-PEG**	45.30 %	Bismuth-dodecanethiol nanoparticles capped with DSPE-PEG	Non-toxic	1
**Bi NP**	27.70 %	Bismuth-dodecanethiol nanoparticles	Non-toxic	2
**Bi-Bi2Se3 NP**	38.10 %	Bismuth-dodecanethiol nanoparticles shelled with Bi_2Se_3	Non-toxic	2
**Sb NP**	44 %	Mesoporous antimony Nanoparticles	Low toxicity up to 100 µg/mL	3
**TiN NP**	49 %	TiN NPs	Non-toxic	4,5
**MoS2 NS**	37.48 %	Hydrothermal MoS_2 nanosheets	Non-toxic	6
**GQD**	62.53 %	Graphene Quantum Dots	Low toxicity below 250 µg/mL	7
**CNDs**	52 %	Carbon NanoDots	Not investigated	8
**CNT**	57.80 %	Lipid-coated Carbon Nanotubes	Low toxicity below 30 µg/mL	9

Table S2: Comparison of the PTCE of different nanoparticles.

REFERENCES

(1) Yu, N.; Wang, Z.; Zhang, J.; Liu, Z.; Zhu, B.; Yu, J.; Zhu, M.; Peng, C.; Chen, Z. Thiol-Capped Bi Nanoparticles as Stable and All-in-One Type Theranostic Nanoagents for Tumor Imaging and Thermoradiotherapy. *Biomaterials* **2018**, *161*, 279–291. https://doi.org/10.1016/j.biomaterials.2018.01.047.

(2) Li, B.; Cheng, Y.; Zheng, R.; Wu, X.; Qi, F.; Wu, Y.; Hu, Y.; Li, X. Improving the Photothermal Therapy Efficacy and Preventing the Surface Oxidation of Bismuth Nanoparticles through the Formation of a Bismuth@bismuth Selenide Heterostructure. *J. Mater. Chem. B* **2020**, *8* (38), 8803–8808. https://doi.org/10.1039/D0TB00825G.

(3) Chen, Y.; Yu, Z.; Zheng, K.; Ren, Y.; Wang, M.; Wu, Q.; Zhou, F.; Liu, C.; Liu, L.; Song, J.; Qu, J. Degradable Mesoporous Semimetal Antimony Nanospheres for Near-Infrared II Multimodal Theranostics. *Nat Commun* **2022**, *13* (1), 539. https://doi.org/10.1038/s41467-021-27835-y.

(4) Karaballi, R. A.; Esfahani Monfared, Y.; Dasog, M. Photothermal Transduction Efficiencies of Plasmonic Group 4 Metal Nitride Nanocrystals. *Langmuir* **2020**, *36* (18), 5058–5064. https://doi.org/10.1021/acs.langmuir.9b03975.

(5) Alvarez, C.; Berrospe-Rodriguez, C.; Wu, C.; Pasek-Allen, J.; Khosla, K.; Bischof, J.; Mangolini, L.; Aguilar, G. Photothermal Heating of Titanium Nitride Nanomaterials for Fast and Uniform Laser Warming of Cryopreserved Biomaterials. *Front. Bioeng. Biotechnol.* **2022**, *10*. https://doi.org/10.3389/fbioe.2022.957481.

(6) Liu, J.; Cui, E.; Zhang, Q.; Xie, D. MoS2-Based Nanocomposites with High Photothermal Conversion Efficiency for Combinational Photothermal/Photodynamic Tumor Therapy. *J. Alloys Compd.* **2024**, *970*, 172489. https://doi.org/10.1016/j.jallcom.2023.172489.

(7) Xuan, Y.; Zhang, R.-Y.; Zhang, X.-S.; An, J.; Cheng, K.; Li, C.; Hou, X.-L.; Zhao, Y.-D. Targeting N-Doped Graphene Quantum Dot with High Photothermal Conversion Efficiency for Dual-Mode Imaging and Therapy in Vitro. *Nanotechnology* **2018**, *29* (35), 355101. https://doi.org/10.1088/1361-6528/aacad0.

(8) Li, D.; Han, D.; Qu, S.-N.; Liu, L.; Jing, P.-T.; Zhou, D.; Ji, W.-Y.; Wang, X.-Y.; Zhang, T.-F.; Shen, D.-Z. Supra-(Carbon Nanodots) with a Strong Visible to near-Infrared Absorption Band and Efficient Photothermal Conversion. *Light Sci Appl* **2016**, *5* (7), e16120–e16120. https://doi.org/10.1038/lsa.2016.120.

(9) Zhao, Y.; Zhao, T.; Cao, Y.; Sun, J.; Zhou, Q.; Chen, H.; Guo, S.; Wang, Y.; Zhen, Y.; Liang, X.-J.; Zhang, S. Temperature-Sensitive Lipid-Coated Carbon Nanotubes for Synergistic Photothermal Therapy and Gene Therapy. *ACS Nano* **2021**, *15* (4), 6517–6529. https://doi.org/10.1021/acsnano.0c08790.
